# Scale-Dependent Habitat Nestedness and Its Implications for Anuran Conservation in the Chengdu Region: A Multi-Extent Analysis

**DOI:** 10.3390/ani14202931

**Published:** 2024-10-11

**Authors:** Xiaoqin Shi, Xiaoke Liu, Youhua Chen

**Affiliations:** 1 Chengdu Institute of Biology, Chinese Academy of Sciences, Chengdu 610041, China; 2 University of Chinese Academy of Sciences, Beijing 100049, China

**Keywords:** anuran amphibians, community assembly, spatial scale, habitat nesting, sampling range

## Abstract

**Simple Summary:**

This study examines the nestedness of anuran species in the Chengdu region, Sichuan, during summers 2019–2020. Using a transect method, the area was surveyed across 23 sites and 8 regions. Habitat data were surveyed with sampling buffers at 1 km, 2 km, and 5 km to assess scale-dependent nestedness. The weighted-nestedness metric (WNODF) revealed strong nested patterns at both site and regional scales. Habitat nestedness was the sole driver, as other factors showed no significant correlation with nestedness. This study recommends habitat diversity protection as a key conservation strategy for anurans in the area.

**Abstract:**

Nestedness in community ecology predicts that species in a species-poor site should be a subset of species of a species-rich site. A variety of ecological mechanisms have been offered to explain community nestedness; however, few studies have systematically discussed the issue of scale dependence when interpreting community nestedness. This study conducted surveys of anuran species data in the vicinity of Chengdu, Sichuan, in the summers of 2019–2020, using the transect method. The study area was divided into 23 sampling sites and 8 regions to explore the relationship between environmental factors and the nested distribution pattern of anuran communities under different sampling extents (with sampling buffers set at 1 km, 2 km, and 5 km). The WNODF (weighted-nestedness metric based on overlap and decreasing fill) results indicated that anurans exhibited a strong nested pattern at both the sampling sites scale and the regional scale. The habitat matrix test results suggested that a small-scale study area requires a correspondingly small habitat-sampling extent to effectively test for habitat nestedness. As the study area expands, the habitat-sampling range can be appropriately increased. The nested pattern of anurans in the vicinity of Chengdu can only be explained by habitat nestedness, as a Spearman’s correlation analysis showed that other environmental factors (area size, connectivity index, concentration index, proximity index, and distance to the city center) were not significantly correlated with the nested sequences of sampling points and regions. Therefore, regarding the conservation strategies for anurans in the vicinity of Chengdu, we recommend prioritizing the protection of areas with higher habitat diversity.

## 1. Introduction

Nestedness is a fundamental ecological concept that predicts the species composition of a species-poor site to be a non-random subset of that of a species-rich site, particularly within fragmented or isolated habitats [1,2,3,4,5,6,7]. This pattern is widely recognized as a key community assembly rule in macroecology, with extensive documentation across diverse systems, including marine and terrestrial islands, mountain ranges, forests, and river systems [8,9,10,11]. This distributional pattern also prevails in microbial communities, although it has not been widely studied until recently [12,13,14]. Prior to this, the nested structure within communities of many species has been extensively studied [6,15,16]. Amphibians, due to their high environmental sensitivity, are also considered to be excellent models for studying community structure [17].

A variety of biological mechanisms can be used to explain community nestedness for different taxonomic groups. The most commonly recognized explanations are the selective extinction hypothesis, the selective colonization hypothesis, passive sampling, habitat nestedness, and disturbances [1,18,19,20,21]. As such, it is interesting to explore the relationship between the single-species or single-site nestedness with other explanatory environmental variables (e.g., habitat diversity). However, research on the community structure of amphibians in the vicinity of Chengdu city and the mechanisms behind it remains relatively scarce.

Scale dependence is a fundamental and pervasive phenomenon in ecological systems [22,23]. This phenomenon can manifest due to various factors, including but not limited to the size of operative sampling units, the extent of the sampling region, and the resolution of data collection [24,25]. Nestedness, a concept derived from species-site ecological matrices, inherently possesses the property of scale dependence [1,5]. Empirical evidence supporting the scale dependence of community nestedness has been documented in several studies. For instance, Cook et al. [1,15] conducted a comprehensive analysis of fish communities in Virginia streams and demonstrated a significant correlation between nestedness and the spatial extent of the sampled area. Similarly, Menegotto et al. [26] provided evidence for scale-dependent effects of sediment on the nested structure of benthic communities. These findings corroborate the theoretical predictions of scale-dependent nestedness patterns [27]. Despite these advancements, there remains a relative paucity of studies specifically investigating the effects of spatial scale on nested patterns across diverse ecological systems. Moreover, a rigorous and systematic evaluation of how scale dependence influences the relationships between nestedness and its associated explanatory predictors has yet to be conducted. This gap in our understanding highlights the need for further research to elucidate the complex interactions between spatial scale, nestedness, and ecological processes [28,29].

In this study, we examined various explanatory variables used to test multiple hypotheses in predicting the community nestedness structure of amphibians in SW China, a biodiversity hotspot. We specifically address two sources of scale dependency: (1) study area extent, which involves varying the spatial boundaries encompassing distinct ecological or geopolitical regions, and (2) spatial sampling range, which refers to the size of the minimum sampling unit. These two factors were analyzed independently and synergistically to assess their impact on community nestedness patterns.

## 2. Materials and Methods

### 2.1. Study Area

Chengdu, a famous tourist attraction and the capital of Sichuan province, is located in southwest China. This region has a subtropical humid monsoon climate, with an average annual precipitation of over 1000 mm, an average annual temperature of 16 °C, and an average elevation of about 500 meters. The terrain of Chengdu slopes from northwest to southeast. Owing to the large vertical height difference, plains, hills, and mountains form a unique geomorphological type within the city, resulting in a variety of land use types and natural environments. Owing to the significant differences in climate, a vertical line in climate zones is formed, and thus a high level of biological diversity can be found in the southwest China [30,31].

**Figure 1:**
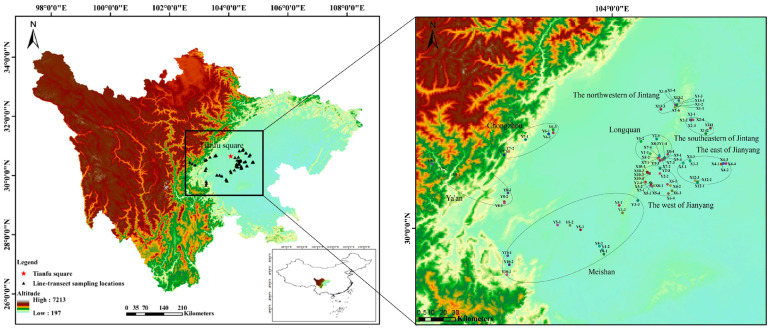
Transect line sampling locations in Sichuan, China (**left**), and eight regions divided (**right**).

A moderate number of sample points were identified from Chengdu and its neighboring urban areas according to the topography, habitat characteristics, altitude, water status, amphibian habits, and distribution characteristics of Chengdu and its surrounding areas. According to the identified sampling points, three sampling lines were selected within a 20 km area, keeping the habitat types as different as possible and separated by more than 1 km. The sampling areas were Jintang County, Jianyang City, Longquanyi District, Meishan City, Chongzhou City, and Ya’an City. The sampling areas were selected based on the following considerations: (1) to use the ecosystem as the background and try to use the administrative division of the county as the unit, but due to the large size of some areas, they are divided into two areas at the regional scale according to the geographical location of the sample; and (2) to select a regional belt with an urbanization gradient of city center–urban area–suburban area–mountain area. Figure 1 shows the 79 sampling lines in Chengdu and surrounding areas, with the red five-pointed star symbolizing the center of Chengdu City, Sichuan Province, i.e., Tianfu Square. The black triangles represent the centroids of the different sampling lines set up in this study, and the black ellipses represent all the sampling lines divided into eight zones at the regional scale.

### 2.2. Amphibians Field Sampling

The transect line method was used to search for amphibians at 23 sites in August 2019 and July 2020. A total of 79 transects were established in 6 counties of the rural region of Chengdu City. The survey was conducted in the breeding seasons. Before the survey, we first downloaded satellite maps of the area from Omap (https://www.ovital.com/, accessed on 10 August 2019 and 20 July 2020) to obtain the basic conditions of the habitat distribution of Chengdu and its surrounding areas. To sample appropriate amphibian habitats such as mountain streams, ponds, paddy fields, and forests, starting at a predetermined location at 20:00, investigators walked 400–600 m at a constant speed of 2 km/h, searching for amphibians in the area within 2 m of the transect line using a strong flashlight. The survey team, consisting of 3 to 5 members, had at least one person recording the species and quantities of amphibians, while the remaining members were responsible for capturing and identifying the amphibians with the assistance of experienced personnel.

### 2.3. Indicative Environmental Variables

**Figure 2:**
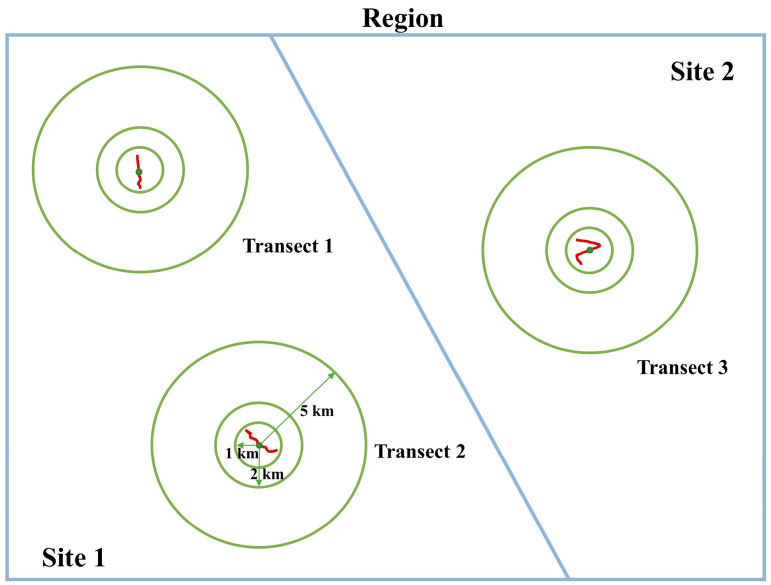
A schematic diagram for evaluating the influence of scale dependence on community structure. In our study, two scale-changing properties were evaluated: varying the study area extent (transect-site-region) and varying the sampling range (1 km, 2 km, and 5 km). Line transects are marked as red polylines.

We have enumerated five widely recognized hypotheses (Table 1), each of which will select one to two indicative environmental variables for analysis. With respect to the analyses concerning the extent of the study area, all species abundance and environmental data were aggregated or combined for each sampling line transect (79 transects), sampling site (23 sites), and sampling region (8 regions) (Figure 1). For environmental covariates at larger spatial scales (i.e., sampling site or sampling region), we averaged all those values extracted for each line transect found within each sampling site or region for a specific sampling range (i.e., a given buffering circle shown in Figure 2 or Figure S1). The buffer zone dimensions, specifically set at 1 km, 2 km, and 5 km, were ascertained based on the migratory capabilities of anuran amphibians [32,33]. Note that here, union operation might be applied, since buffering circles constructed for different line transects might overlap (Figure S2).

For each sample site and area with different sampling ranges, six habitat variables that may influence amphibian distribution patterns were collected: habitat area, connectivity index (CONN), concentration index (CONC), proximity index (PROX), and distance to the city center (DCC) [35,36,37]. The habitat data source is 30 m land cover data in 2020 (GLC_FCS30); all habitat types in the final study sample sites and regions were grouped into 16 categories (Table S1 or Table S2). Based on the habitat data, the amphibian-unsuitable habitats were excluded according to a radius of 1 km, 2 km, and 5 km, and the suitable habitat area size and other parameters were calculated for each sampling site and region.

The following three indices were calculated for each sampling site (23 sites) and each sampling region (8 regions). A $\left(i\right)$ represented the area of patch i; $A\left(j\right)$ represented the area of patch j (patch *i* ≠ patch *j*); $d_{ij}$ represented the distance between focal patch i and patch j [35].

The connectivity index measures the migration potential of a species, a measure of the continuity between spatial structural units of the landscape associated with the characteristic scale of the subject or process of study. A high index value indicates high CONN between sites or regions. The formula was as follows:

(1) $$CONN(i)=\sum_{j\neq i}A\left(j\right)\times e^{-d_{ij}}.$$

The concentration index measures the aggregation of suitable habitats in a focal grid cell and its four nearest neighbors. The formula was as follows:

(2) $$CONC\left(i\right)=\sum_{j\in s}A\left(j\right).$$

The proximity index (PROX) measures the distance between two cells through calculating area size of suitable habitat of a focal patch and the distances to patches around a focal patch. The formula was as follows:

(3) $$PROX\left(i\right)=\sum_{j\neq i}\frac{A\left(i\right)+A\left(j\right)}{d_{ij}^{2}}.$$

Distance to the city center (DCC) is simply the Euclidean distance to the city center (Tianfu Square of Chengdu City), representing the degree of human disturbance. The rationale for selecting the DCC is based on the following considerations: Firstly, DCC is a reliable indicator of human influence, as urban areas are typically centers of human activity. Secondly, it is a quantifiable and objective measure that enables standardization across different study sites. We also computed DCCs for each sampling site (23 sites) and each sampling region (8 regions).

### 2.4. Nestedness Metrics

The WNODF (weighted-nestedness metric based on overlap and decreasing fill) was used to calculate the nested-amphibian-community distribution patterns [38]. This index has been shown to be effective in reducing or avoiding type I errors and is currently the best nested index for calculating species-richness matrices [38,39]. For a matrix with *n* columns and m rows, the weighted-nestedness overlap and decreasing-fill (WNODFc) value for column *j* represents the percentage of cells in column j that have lower abundance values than the corresponding cells in column *i*. The calculation of pairwise nestedness values along the rows is similar (i.e., WNODFr). The formula was as follows:

(4) $$\left\{\begin{array}{c} \mathrm{WNODFc}=100\sum_{i=1}^{n-1}\sum_{j=i+1}^{n}\frac{K_{ij}}{N_{j}}\\ \mathrm{WNODFr}=100\sum_{i=1}^{m-1}\sum_{j=i+1}^{m}\frac{L_{ij}}{M_{j}} \end{array}\right.$$

(5) $$WNODF=\frac{2(WNODFc+WNODFr)}{m\left(m-1\right)+n(n-1)}$$

*K_ij_* represented the number of cells with lower abundance in column *j*, and *N_i_* represented the total number of non-empty cells in column *i*. As a comparison, *L_ij_* represented the number of cells with lower abundances in row *j*, and *M_j_* represented the total number of non-empty cells in row *i*.

The above calculations were performed using an R 4.2.2 bipartite package [40], and the statistics were estimated with 95% confidence intervals using a Fixed–fixed null model with 1000 randomly generated mats [41]. We also employed an R MBI package [42] to calculate the contribution of each sampling site and region to the overall nested pattern, denoted as MLCN (a local site contribution to overall row nestedness). In subsequent impact analyses, the relationship between this nestedness index and environmental factors will be incorporated, with the aim of identifying which sampling points play a critical role in forming the overall nested pattern.

### 2.5. Explanatory Analyses

The species location matrix was rearranged by WNODF calculation to obtain a maximally aligned nested matrix, and the nested sequences of the sampling sites and regions were analyzed by Spearman correlation with habitat variables that may affect the nested-distribution pattern of anuran amphibian communities [43]. To prevent an increase in Type I error due to the multiple comparisons conducted in these correlation tests, a Bonferroni correction was implemented to confirm the statistical significance of each correlation. For testing the selective extinction hypothesis, we utilized the CONC in 1 km, 2 km, and 5 km circles and the habitat area metrics of different sampling scales from the land use dataset in 2020 as the indicative variables. For testing the selective colonization hypothesis, we utilized CONN and PROX in 1 km, 2 km, and 5 km circles as the indicative variables.

We used the random-placement model to examine whether the passive sampling could explain the nestedness pattern of amphibian communities [44]. According to this model, the expected value $S\left(\alpha \right)$ of the species based on the *k*-th island of area of A is calculated. $S\left(\alpha \right)$ represents the total number of species; α represents a relative area size a; $n_{i}$ represents the abundances *n*_1_, *n*_2_, …, *n_s_* of the S species; and $\sigma^{2}$ is the variance of $S\left(\alpha \right)$. The hypothesis of random distribution should be rejected if more than one-third of the points lie outside one standard deviation of the expected curve, and if the points are not evenly distributed.

(6) $$S\left(\alpha \right)=S-\sum_{i=1}^{S}{\left(1-a\right)}^{n_{i}}$$

where $\alpha =a_{k}/\sum_{k=1}^{K}a_{k}$ and $\sigma^{2}=\sum_{i=1}^{S}{\left(1-\alpha \right)}^{n_{i}}-\sum_{i=1}^{S}{\left(1-\alpha \right)}^{2n_{i}}$. $a_{k}$ is the size of suitable habitats within a specific buffering circle (1 km, 2 km or 5 km) for a specific line transect or the area size of combined suitable habitats (i.e., union) for different line transects within a sampling site or region for a specific buffering circle (1 km, 2 km or 5 km).

For testing the habitat nestedness hypothesis, we used the R 4.2.2 bipartite package to directly test whether the habitat location matrix (for the matrix, see Tables S2 and S3) conforms to a nested-distribution pattern. If the sample sites and regional habitat matrices were significantly nested, then the anuran amphibian communities around Chengdu city conformed to the habitat-nesting hypothesis. For testing the human disturbance, we utilized the distance from each sampling line transect, the center of each sampling site, or the center of sampling region to the city center (namely DCC).

## 3. Results

### 3.1. Species Composition and Nested-Distribution Patterns of Anuran Amphibians

A total of 820 amphibian individuals belonging to 17 species in 12 genera, 6 families, and 1 order were captured during the two surveys in August 2019 and July 2020. Ranidae had the largest number of species recorded, with 10 species accounting for 58.8% of the total species (Table S3). This was followed by two families, Dicroglossidae and Microhylidae, each of which accounted for 11.8% of the total species (Table S3). The least common species were in the families Bufonidae, Megophryidae, and Rhacophoridae, each accounting for 5.9% of the total species (Table S3). The largest number of species was from the genus *Rana*, with five species, accounting for 29.4% of the total species, and other genera in the Ranidae were represented by single species.

The WNODF calculations showed that the anuran amphibian communities around Chengdu were significantly nested, both at the sample-site level (WNODF = 28.85, *p* < 0.001) and at the regional level (WNODF = 37.31, *p* < 0.001), and the nestedness at the regional scale was improved compared to the nestedness at the sample sites scale (Table 2). At the sample sites level, there was a significant nested pattern of species (WNODFr = 19.43, *p* < 0.001) and locations (WNODFc = 33.32, *p* < 0.001) of anuran amphibians (Table 2). At the regional level, anuran amphibians showed a significant nested pattern of species (WNODFr = 39.61, *p* < 0.001) and locations (WNODFc = 49.49, *p* < 0.001) (Table 2).

### 3.2. Relationships between Environmental Variables and Nestedness across Sampling Sizes and Regions

The range of habitat types extracted had implications for habitat nestedness in anuran amphibians, with relatively small ranges, might provide more favorable evidence (Table 2). At the 23-sample-sites scale, habitat types originating within the 1 km buffer have a significant nested structure (NODF = 80.74, *p* < 0.001), but habitat types within the 2 km and 5 km buffers did not exhibit nestedness. At the 8-sample-regions scale, habitat types originating within the 1 km (NODF = 73.55, *p* < 0.001) and 2 km (NODF = 78.28, *p* < 0.001) buffer have a significant nested structure.

**Figure 3:**
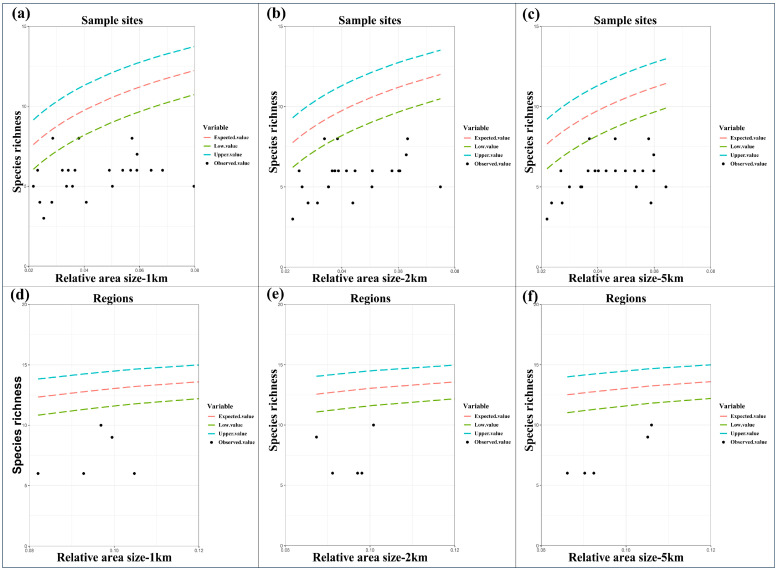
The relationships between species richness and relative area size predicted by the random placement model at sample-site (see subfigures (**a**–**c**)) and regional spatial scales (see subfigures (**d**–**f**)). Expected values and the 95% confidence interval (dashed lines), observed species richness of amphibians (black dots). Note that here, suitable habitat area sizes were determined by using 1 km, 2 km, and 5 km buffering circles.

The results of the random placement model showed that the actual observed richness of anuran amphibian communities around Chengdu was outside the 95% confidence interval of the prediction curve (Figure 3) at both the sample sites and the regional scale, so the passive sampling hypothesis was rejected.

The Spearman’s correlation analysis showed that the nested distribution pattern of anuran amphibians around Chengdu was not significantly correlated with the suitable area and concentration index (CONC) and thus did not conform to the selective extinction hypothesis. The selective colonization hypothesis was also rejected as neither connectivity index (CONN) nor the proximity index (PROX) showed significance. In addition, the distance to the city center (DCC) did not show significance, suggesting that the human disturbance hypothesis is not relevant to the nested pattern of anuran amphibians in the study area (Table 3). Finally, for each site’s contribution to overall nestedness (MLCN), no environmental factors exhibited significance, indicating that the contribution of each site to the overall nestedness is also not influenced by the environmental factors presented in Table 3.

## 4. Discussion

Multiple studies have demonstrated the presence of nesting patterns among amphibians across various island, pond, and riverine habitats, primarily attributed to their limited dispersal capabilities between habitat patches [17,45,46]. Our findings indicate that nestedness is a significant characteristic of anuran amphibians in the region surrounding Chengdu, Sichuan. Furthermore, by subdividing the sampling range, we revealed that nestedness in anuran amphibians is prevalent across different habitat patch scales. Although few studies have specifically addressed the influence of spatial scale on nesting patterns, Cook et al. [15] observed that nesting in fish was significantly correlated with the spatial extent of the sampled area. However, their study primarily focused on the definition of sampling boundaries rather than variations in spatial scale. In contrast, our research demonstrates that variations in sampling-patch size exert a tangible impact on nestedness: as the sampling area expands, the nestedness of anuran amphibians may increase. Variations in spatial scale exert significant influence on patterns of biodiversity, primarily attributable to the differential contributions of local and regional processes, as well as their interplay with the dispersal of organisms [47]. In summary, our findings indicate that the scale of sampling is a contributing factor to understanding the nestedness of amphibian communities. This underscores the significance of considering spatial scale in ecological research to accurately interpret patterns of species distribution.

The nested distribution of anurans around Chengdu, Sichuan, is most likely related to the nested pattern of habitats, which is in line with the habitat nestedness hypothesis. In fact, there is substantial literature that demonstrates the nested pattern of amphibians can be explained by habitat nesting [17,48,49]. This may be because the majority of amphibian species have a preference for a certain type of habitat, and habitats near water bodies are not randomly distributed but exhibit a nested distribution; ultimately, the nested distribution of habitats leads to the nested distribution of species [49]. Additionally, we discovered that the nested structure of amphibian habitats is associated with the sampling extent of habitat types extracted. Our findings demonstrate that at the scale of 23 sampling points, when the habitat-sampling extent is 1 km, the habitats exhibit significant nestedness. Furthermore, at the scale of 8 regional areas, when the habitat-sampling extent is both 1 km and 2 km, the habitats display a significant nested structure. We believe that the specific preferences of amphibians for habitats that are conducive to reproduction, foraging, or hiding are key factors. If the sampling scope of the habitat is too broad, capturing habitats beyond the range preferred by amphibians could increase the complexity and variability of habitat types, thereby potentially losing the significance of the habitat’s nested structure. However, as the scope of the research area expands, the number of habitat types between regions increases compared to the site’s habitat types, allowing for a corresponding broadening of the sampling breadth of habitat types.

In summary, our study showed that there was strong evidence of scale dependence of the underlying mechanisms explaining taxonomic nestedness of the amphibian community. However, we did not find that alternative hypotheses were important (e.g., the selective extinction hypothesis and the environmental heterogeneity hypothesis).

In contrast to other studies [17,46,50], our study did not reveal that the nested distribution pattern of anuran amphibians in the vicinity of Chengdu, Sichuan, aligns with the selective extinction hypothesis, as neither the area factor nor the habitat aggregation index demonstrated significance at the sample sites and regional scales. The selective extinction hypothesis posits that species with larger minimum area requirements and smaller population sizes are at an increased risk of extinction. In this study, the anuran amphibian species found in different regions do not exhibit significant differences in survival agility, habitat specificity, geographic range size, and area requirements, thus not supporting the selective extinction hypothesis [6].

The connectivity index (CONN) and proximity index (PROX) showed a not-significant correlation with the nestedness of anuran amphibians, indicating that the selective colonization hypothesis does not lead to the nested pattern of anuran amphibians. The selective colonization hypothesis emphasizes that species with enhanced dispersal abilities can preferentially occupy a greater number of viable habitats. Species with robust dispersal capabilities, such as birds and mammalian taxa, contribute to the formation of nested community structures through their dispersal and colonization processes [51,52,53]. Nonetheless, studies have suggested that the frequent dispersal of species may also attenuate nestedness by mitigating the effects of differential colonization, which is achieved by reducing extinction rates [54,55] (Wang et al., 2024; Dardanelli and Bellis, 2021). However, the dispersal and settlement abilities of amphibians are generally inferior [56], which may impede their ability to spread to more distant habitats, thereby diminishing the potential for nestedness to be fostered by dispersal mechanisms.

Excluding other environmental factors from consideration, our research findings indicated that there was a significant relationship (*p* < 0.05, which is the *p*-value adjusted before Bonferroni correction) between the connectivity index (CONN) and a local site contribution to overall nestedness (MLCN) of the 23 sampling sites (Table 3). This suggested that, at the sampling-sites scale, habitats with lower connectivity contribute more to the nested pattern of anurans. Studies have indicated that the short-term impacts of habitat loss and fragmentation increase with enhanced dispersal ability [57], underscoring the critical importance of habitats with low connectivity for species that are less capable of dispersal. This study also demonstrated this conclusion from the perspective of nested structures.

The passive sampling hypothesis emphasizes that the sampling mechanism of community nestedness, which is caused by the differences in the size of the sampling area, has nothing to do with the real distribution of the species in nature and tends to accelerate the generation of nestedness [58]. Usually, the passive sampling hypothesis has served as a null model and had been rarely supported in the literature as an influencing factor that can drive taxonomic nestedness or other ecological patterns [6,7,59,60,61,62]. However, in our study, we found weak evidence of passive sampling in structuring amphibian community nestedness.

Human disturbance includes habitat change and loss, increase in road traffic, agricultural intensification, and urbanization. One study suggested that human disturbance could affect nestedness in Great Basin butterfly communities [63]; however, there is little field evidence that human disturbance can affect the nestedness of vertebrate communities. Of course, human disturbance may promote the formation of nestedness when interacting with other factors such as fragment size and habitat structure at the community and individual species levels and species’ habitat requirements. Apparently, species with large area requirements will appear in large patches, while species with small area requirements might be found in more places. The variation in the habitat or area requirement of species might be in conflict with human activities and infrastructure construction in different rural areas. The human disturbance hypothesis was not supported in our study, because the variable DCC was not significantly and positively correlated with nestedness across different sampling scales. Additionally, it should be noted that we have recorded the invasive species Lithobates catesbeianus once in Jintang and Jianyang, which can also be considered a consequence of human activities. Given the limitations of our data, we have not explored the changes in the nested pattern before and after the invasion. Therefore, the profound impact of invasive species on the nested pattern of native species still requires further research.

It is important to note that, while we have confirmed the presence of nestedness in amphibian communities, we must acknowledge that the elusive nature of amphibians during surveys may lead to an underestimation of the number of species captured by the transect method. However, our study was conducted during the breeding season and at night, when amphibians are most active, which helps to mitigate some of this potential bias. Nevertheless, we still recommend employing a variety of capture techniques to sample all species present in the community [64].

The scale of examination has been recognized as a critical issue in ecology [22,23,65]. The present study illustrates the importance of scale in community structure. In the process of examining habitat nestedness, we found that as the study area expands from the point scale to the regional scale, changes in the spatial sampling breadth of habitats can also affect the interpretability and significance level of habitat nestedness. One explanation for scale dependence being pervasive was that when the scale changed (regardless of sampling grain size or study extent), additional environmental heterogeneity was introduced. These phenomena can drive the relationship between nestedness and the underlying environmental covariates to become more complex. Our results highlighted that the scale dependence of amphibian community-level and individual site-level nestedness patterns resulting from either varying the study extent, the sampling grain size, or both, can lead to a significant relationship between indicative variables and specific nestedness metrics (Table 2). Despite other environmental factors showing no significance when the regional and sampling scope vary, this may be because these environmental factors are incapable of explaining the nestedness of anurans within the order Anura. However, we still recommend that in future studies, the appropriate range of environmental factors sampling should be determined based on the size of the research scope.

## 5. Conclusions

Our study demonstrated that it was necessary and important to carry out multi-scale analyses when testing multiple hypotheses for the driving factors of ecological community structure. Moreover, our study emphasized that the multi-scale analyses should consider different mechanisms driving scale dependence (e.g., study area extent and sampling range as extensively studied here). Finally, we propose that for the conservation of anurans, maintaining habitat diversity is of paramount importance, and our results might have impacts on the management of rural area biodiversity and improvement of protection efforts. The evaluation of nestedness in different regional gradients can help people to better understand the impact of urbanization on amphibian community structure and provide a theoretical basis for the formulation of rural amphibian diversity protection strategies.

## Figures and Tables

**Table 1:** Potential hypotheses being tested for explaining the community nestedness pattern of amphibian communities in rural regions of Chengdu City, Sichuan, China. Full names of variables can be referred to the main text.

Hypothesis	Definition	Main References
The selective extinction hypothesis	Species with large minimum area requirement will extinct first and the selective extinction of species will lead to nestedness.	[5,34]
The selective colonization hypothesis	Species or populations are able to selectively colonize and reproduce in specific environments, emphasizing that species are adaptive and selective to their environments, and that species with greater dispersal capabilities are able to preferentially occupy more sites for survival.	[18]
The passive sampling hypothesis	The possibility of sampling more the more abundant species.	[19]
Habitat nestedness hypothesis	The nested nature of species distributions may be due to the preference of different species for specific habitat conditions.	[20]
Human disturbance	In a fragmented landscape, with different levels of human disturbance and with species having different tolerance to disturbance, extinction would increase and colonization would decrease in highly disturbed fragments. These processes are expected to decrease the size of species subsets with increasing disturbance, giving rise to a nested pattern.	[21]

**Table 2:** Results of the nestedness analysis of the anuran amphibian community abundance site matrix and the habitat-site matrix with different sampling extents in the surrounding areas of Chengdu, Sichuan.

Nestedness Analyses of Species Abundance	Anurans (Sample-Site)			Anurans (Sample-Region)					
	Observed	Expected	*p*	Observed	Expected	*p*			
NODF	54.77	17.22	<0.001	66.92	26.99	<0.001			
NODFc	58.18	12.03	<0.001	82.24	15.49	<0.001			
NODFr	47.6	13.7	<0.001	69.82	24.82	<0.001			
WNODF	28.85	54.43	<0.001	37.31	57.64	<0.001			
WNODFc	33.32	41.59	<0.001	49.49	50.26	<0.001			
WNODFr	19.43	45.72	<0.001	39.61	56.24	<0.001			
**Nestedness Analyses of Habitat**	**Habitat in Sample-Site (1 km)**			**Habitat in Sample-Site (2 km)**			**Habitat in Sample-Site (5 km)**		
	**Observed**	**Expected **	***p***	**Observed**	**Expected **	***p***	**Observed**	**Expected **	***p***
NODF	80.74	79.20	<0.001	83.44	83.13	0.22	72.86	73.34	0.988
NODFc	81.09	80.06	<0.001	82.22	82.21	0.52	72.65	72.84	0.842
NODFr	79.88	77.37	<0.001	86.03	85.08	0.12	73.31	74.40	0.999
	**Habitat in Regional Scale (1 km)**			**Habitat in Regional Scale (2 km)**			**Habitat in Regional Scale (5 km)**		
	**Observed**	**Expected **	***p***	**Observed**	**Expected **	***p***	**Observed**	**Expected **	***p***
NODF	73.55	78.75	<0.001	78.28	73.72	<0.001	60.69	60.91	0.76
NODFc	84.09	86.48	<0.001	86.86	81.99	<0.001	78.66	78.57	0.39
NODFr	69.44	76.95	<0.001	75.64	71.79	<0.001	56.50	56.79	0.86

**Table 3:** Spearman relationships between environmental factors collected from different sampling ranges and community structure indices at the sample-sites and regions.

	Sample-Sites				Sample-Regions			
	MNODF		MLCN		MNODF		MLCN	
	Coefficient	*p*	Coefficient	*p*	Coefficient	*p*	Coefficient	*p*
Area 1 km	0.291	0.179	0.174	0.428	−0.619	0.102	−0.31	0.456
Area 2 km	0.287	0.201	0.201	0.357	−0.333	0.42	−0.357	0.385
Area 5 km	0.349	0.103	0.185	0.398	−0.333	0.42	−0.167	0.693
PROX 1 km	−0.2	0.361	−0.296	0.17	−0.548	0.16	−0.571	0.139
PROX 2 km	−0.231	0.288	−0.296	0.17	−0.548	0.16	−0.571	0.139
PROX 5 km	−0.208	0.34	−0.321	0.135	−0.548	0.16	−0.571	0.139
CONC 1 km	−0.35	0.102	0.02	0.929	0.619	0.102	0.31	0.456
CONC 2 km	−0.357	0.095	0.013	0.955	0.452	0.26	0.19	0.651
CONC 5 km	−0.336	0.117	−0.007	0.975	−0.333	0.42	0.167	0.693
CONN 1 km	−0.277	0.201	−0.467	0.025	−0.214	0.61	−0.405	0.32
CONN 2 km	−0.264	0.224	−0.454	0.029	−0.5	0.207	−0.405	0.32
CONN 5 km	−0.246	0.258	−0.49	0.018	−0.571	0.139	−0.143	0.736
DCC	0.138	0.529	0.076	0.729	−0.333	0.42	0.095	0.823

## Data Availability

The datasets generated and/or analyzed during the current study are available upon request to the corresponding author.

## Supplementary Materials

The following supporting information can be downloaded at: https://www.mdpi.com/article/10.3390/ani14202931/s1, Figure S1: Different sampling grain sizes used for the present study. We used the center of each line transect as the center for drawing the buffering circles with radius of 0 km, 1 km, 2 km and 5 km, respectively. Figure S2: Union operation when two buffering circles were intersected when conducting explanatory analyses of environmental variables and nestedness at sampling-site or regional extents. Habitat or land use types inside the intersected zone as illustrated in the figure will be counted for one time only. Table S1: The species number of individuals of amphibians registered around Chengdu, Sichuan, China in 2019 and 2020. Table S2: Matrix of habitat locations within different sampling buffers at 23 sampling sites around Chengdu city, with sampling ranges of 1, 2 and 5 in (km). Table S3: Matrix of habitat locations within different sampling buffers at 8 sampling regions around Chengdu city, with sampling ranges of 1, 2 and 5 in (km).
