# Supplementary material for: Scale-Dependent Habitat Nestedness and Its Implications for Anuran Conservation in the Chengdu Region: A Multi-Extent Analysis
Source: Animals (Basel). 2024 Oct 11;14(20):2931. doi: 10.3390/ani14202931 (PMC11503938; doi:10.3390/ani14202931)
Supplement: Supplementary file 1 [file animals-14-02931-s001.zip › animals-3226344-supplementary.pdf]

Supporting Information

Figures and tables

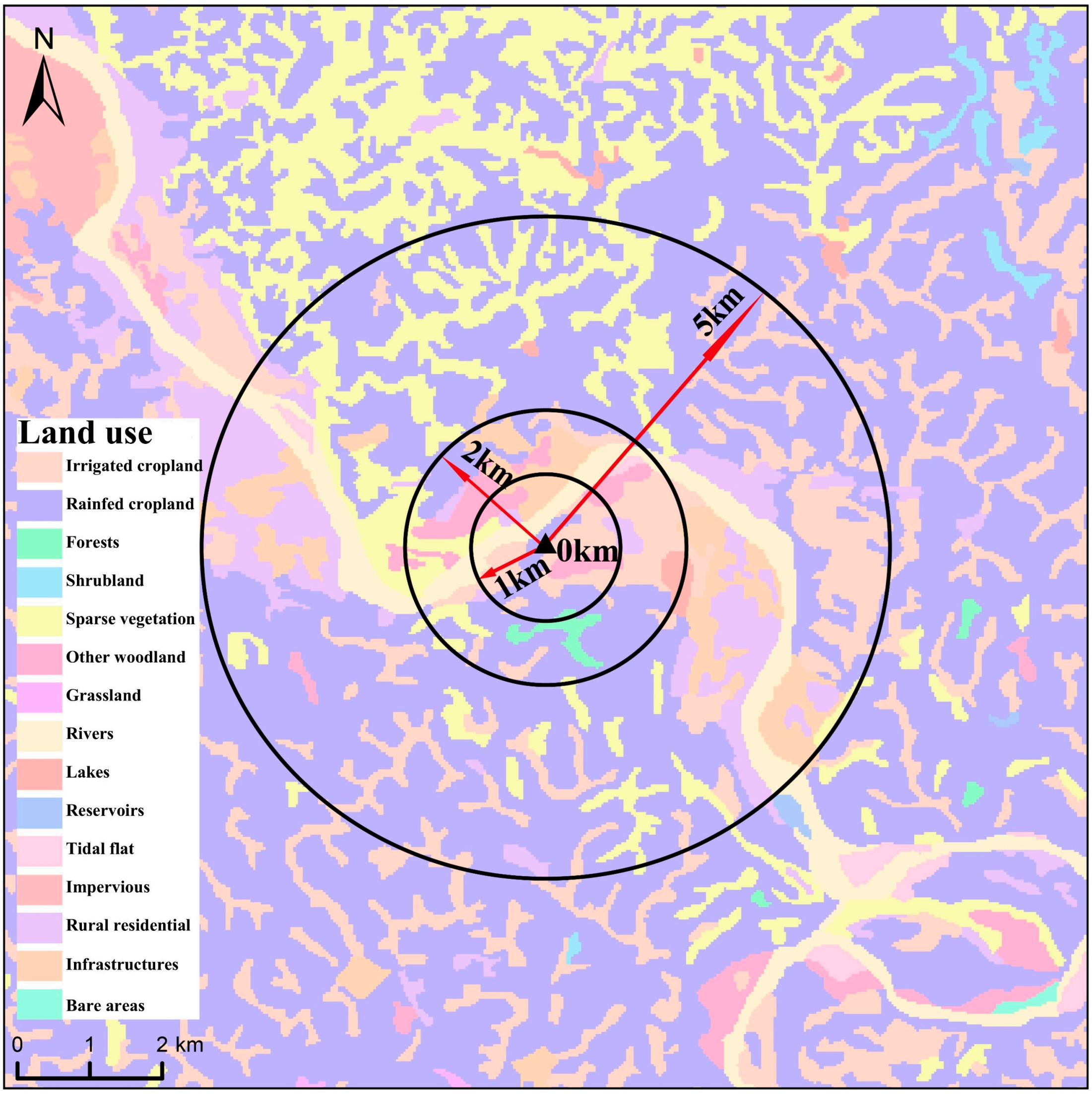

Figure S1. Different sampling grain sizes used for the present study. We used the center of each line transect as the center for drawing the buffering circles with radius of 0 km, 1 km, 2 km and 5 km, respectively.

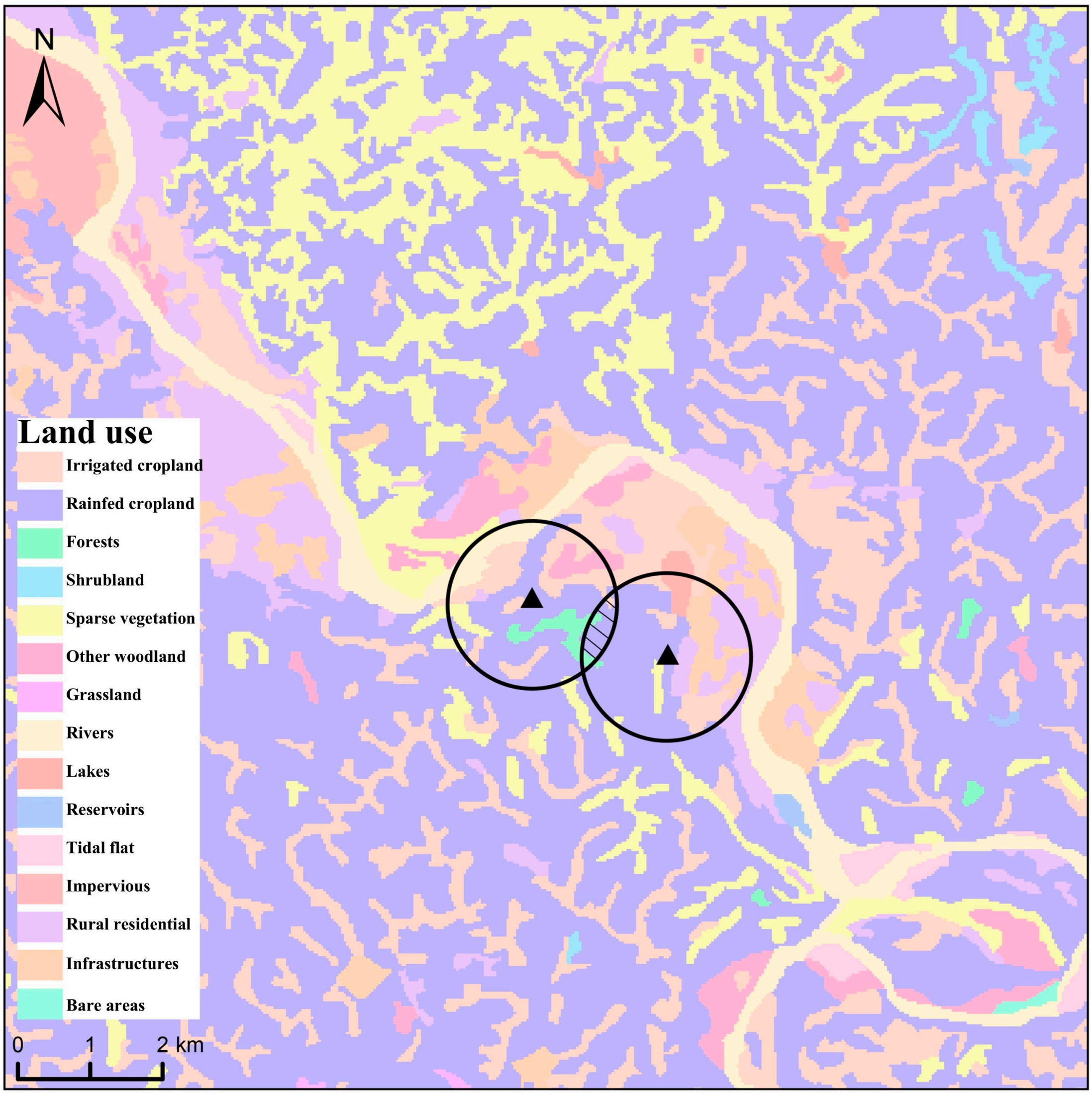

Figure S2. Union operation when two buffering circles were intersected when conducting explanatory analyses of environmental variables and nestedness at sampling-site or regional extents. Habitat or land use types inside the intersected zone as illustrated in the figure will be counted for one time only.

Table S1. Matrix of habitat locations within different sampling buffers at 23 sampling sites around Chengdu city, with sampling ranges of 1, 2 and 5 in (km).

Sample -sites code	Rainfed cropland			Herbaceous cover			Irrigated cropland			Open evergreen broadleaved forest			Closed evergreen broadleaved forest			Open deciduous broadleaved forest			Closed deciduous broadleaved forest			Open evergreen needle-leaved forest		
	1	2	5	1	2	5	1	2	5	1	2	5	1	2	5	1	2	5	1	2	5	1	2	5
X1	1	1	1	1	1	1	1	1	1	0	0	1	1	1	1	0	0	0	1	1	1	0	1	1
X2	1	1	1	0	0	1	1	1	1	0	1	1	0	1	1	0	0	0	0	0	1	0	0	1
X3	1	1	1	0	0	1	1	1	1	0	1	1	0	1	1	0	0	1	0	1	1	0	1	1
X4	1	1	1	0	1	1	1	1	1	1	1	1	1	1	1	0	0	0	0	1	1	1	1	1
X5	1	1	1	0	0	1	1	1	1	1	1	1	1	1	1	0	0	0	0	0	1	1	1	1
X6	1	1	1	0	0	1	1	1	1	0	1	1	0	0	1	0	0	0	0	0	0	0	0	1
X7	1	1	1	1	1	1	1	1	1	0	0	1	1	1	1	0	0	0	1	1	1	0	1	1
X8	1	1	1	0	0	1	1	1	1	1	1	1	1	1	1	0	0	0	0	0	1	1	1	1
X9	1	1	1	0	1	1	1	1	1	1	1	1	1	1	1	0	0	0	1	1	1	1	1	1
X10	1	1	1	1	1	1	1	1	1	1	1	1	1	1	1	0	0	0	1	1	1	1	1	1
X12	1	1	1	0	0	1	1	1	1	0	0	1	0	0	1	0	0	0	0	0	0	0	0	1
X13	1	1	1	0	0	1	1	1	1	0	1	1	0	0	1	0	0	0	0	1	1	0	0	1
X14	1	1	1	0	0	1	1	1	1	1	1	1	1	1	1	0	0	0	1	1	1	1	1	1
Y1	1	1	1	0	1	1	1	1	1	1	1	1	1	1	1	0	0	0	1	1	1	1	1	1
Y2	1	1	1	0	0	1	1	1	1	0	1	1	1	1	1	0	0	0	0	1	1	0	1	1
Y3	1	1	1	0	1	1	1	1	1	1	1	1	1	1	1	0	0	0	0	1	1	1	1	1
Y4	1	1	1	1	1	1	1	1	1	1	1	1	1	1	1	0	1	1	1	1	1	1	1	1
Y5	1	1	1	0	1	1	1	1	1	0	0	1	0	0	1	0	0	0	0	0	1	0	0	0

Y6	1	1	1	0	1	1	1	1	1	1	1	1	1	1	1	0	0	0	1	1	1	0	1	1
Y7	1	1	1	0	0	0	1	1	1	0	1	1	1	1	1	1	1	1	1	1	1	1	1	1
Y8	1	1	1	0	1	1	1	1	1	0	1	1	1	1	1	0	0	0	1	1	1	0	1	1
Y9	1	1	1	1	1	1	1	1	1	0	0	0	1	1	1	0	0	0	1	1	1	1	1	1
Y10	1	1	1	1	1	1	1	0	1	1	1	1	1	1	1	0	0	1	0	1	1	0	1	1

Sample-sites code	Closed evergreen needle-leaved forest			Shrubland			Evergreen shrubland			Grassland			Wetlands			Impervious surtaces			Bare areas			Water body		
	1	2	5	1	2	5	1	2	5	1	2	5	1	2	5	1	2	5	1	2	5	1	2	5
X1	1	1	1	0	0	0	0	0	0	1	1	1	0	0	1	1	1	1	0	0	1	0	0	1
X2	0	0	1	0	0	0	0	0	0	0	1	1	0	0	0	1	1	1	0	0	1	1	1	1
X3	0	1	1	0	0	0	0	1	1	0	1	1	0	0	0	1	1	1	0	0	0	1	1	1
X4	1	1	1	0	0	0	0	0	0	1	1	1	1	1	1	1	1	1	0	0	0	1	1	1
X5	1	1	1	0	0	0	0	0	0	1	1	1	0	0	0	1	1	1	0	0	0	1	1	1
X6	0	0	1	0	0	0	0	0	0	1	1	1	0	0	1	1	1	1	0	0	0	0	1	1
X7	1	1	1	0	0	0	0	0	0	1	1	1	0	0	0	1	1	1	0	0	0	0	0	1
X8	1	1	1	0	0	0	0	0	0	1	1	1	0	0	0	1	1	1	0	0	0	1	1	1
X9	1	1	1	0	0	0	0	0	0	1	1	1	0	0	0	1	1	1	0	0	0	1	1	1
X10	1	1	1	0	0	0	0	0	0	1	1	1	0	0	0	1	1	1	0	0	0	1	1	1
X12	1	1	1	0	0	0	0	0	0	0	1	1	0	0	0	1	1	1	0	0	0	0	1	1
X13	0	0	1	0	0	0	0	0	0	1	1	1	0	0	1	1	1	1	0	0	1	1	1	1
X14	1	1	1	0	0	0	0	0	0	1	1	1	0	0	0	1	1	1	0	0	0	1	1	1
Y1	1	1	1	0	0	0	0	0	0	1	1	1	0	0	0	1	1	1	0	0	0	0	0	1
Y2	1	1	1	0	0	0	0	0	0	1	1	1	0	0	0	1	1	1	0	0	0	0	1	1
Y3	1	1	1	0	0	0	1	1	1	1	1	1	0	0	0	1	1	1	0	0	0	0	0	1

Y4	1	1	1	1	1	1	1	1	1	1	1	1	0	0	0	1	1	1	0	0	0	1	1	1
Y5	0	1	1	0	0	0	0	0	0	1	1	1	0	0	1	1	1	1	0	0	1	1	1	1
Y6	1	1	1	0	0	1	0	1	1	1	1	1	0	0	0	1	1	1	0	0	0	0	1	1
Y7	1	1	1	0	0	1	0	1	0	1	1	1	0	0	0	1	1	1	0	0	0	0	1	1
Y8	1	1	1	0	0	0	0	0	0	0	1	1	0	0	1	1	1	1	0	0	0	0	1	1
Y9	1	1	1	0	0	0	0	0	0	1	1	0	0	0	0	1	1	1	0	1	1	1	1	1
Y10	1	1	1	0	0	1	0	0	0	1	1	1	0	0	0	1	1	1	0	0	0	1	1	1

Table S2. Matrix of habitat locations within different sampling buffers at 8 sampling regions around Chengdu city, with sampling ranges of 1, 2 and 5 in (km).

Sample-sites code	Rainfed cropland			Herbaceous cover			Irrigated cropland			Open evergreen broadleaved forest			Closed evergreen broadleaved forest			Open deciduous broadleaved forest			Closed deciduous broadleaved forest			Open evergreen needle-leaved forest		
	1	2	5	1	2	5	1	2	5	1	2	5	1	2	5	1	2	5	1	2	5	1	2	5
Ya'an	1	1	1	1	1	1	1	1	1	0	0	0	1	1	1	0	0	1	1	1	1	1	1	1
Meishan	1	1	1	1	1	1	1	1	1	0	1	1	1	1	1	0	0	1	0	0	1	0	0	1
Longquanyi	1	1	1	1	1	1	1	1	1	1	1	1	1	1	1	0	0	0	1	1	1	1	1	1
Jintang Southeast	1	1	1	1	1	1	1	1	1	1	1	1	0	1	1	0	0	1	0	1	1	0	1	1
Jintang Northwest	1	1	1	1	1	1	1	1	1	1	1	1	1	1	1	0	0	1	1	1	1	0	1	1
Jianyang West	1	1	1	0	1	1	1	1	1	1	1	1	1	1	1	0	0	0	0	1	1	1	1	1
Jenyang East	1	1	1	1	1	1	1	1	1	1	0	1	1	1	1	0	0	0	0	0	1	1	1	1
Chongzhou	1	1	1	1	1	1	1	1	1	1	1	1	1	1	1	0	0	1	1	1	1	1	1	1

Sample-sites code	Closed evergreen needle-leaved forest	Shrubland	Evergreen shrubland	Grassland	Wetlands	Impervious surtaces	Bare areas	Water body

	1	2	5	1	2	5	1	2	5	1	2	5	1	2	5	1	2	5	1	2	5	1	2	5
Ya'an	1	1	1	0	0	1	1	1	1	1	1	1	0	0	0	1	1	1	0	0	0	0	0	1
Meishan	1	1	1	0	0	0	0	0	0	1	1	1	0	0	1	1	1	1	0	0	1	0	1	1
Longquanyi	1	1	1	0	0	0	0	0	0	1	1	1	0	0	0	1	1	1	0	0	1	1	1	1
Jintang Southeast	0	1	1	0	0	0	0	0	0	1	1	1	0	0	0	1	1	1	0	1	1	0	1	1
Jintang Northwest	1	1	1	0	0	0	0	0	0	1	1	1	0	1	1	1	1	1	0	1	1	1	1	1
Jianyang West	1	1	1	0	0	0	0	0	0	1	1	1	0	0	0	1	1	1	0	0	1	1	1	1
Jenyang East	1	1	1	0	0	0	0	0	0	1	1	1	0	0	0	1	1	1	0	0	0	1	1	1
Chongzhou	1	1	1	0	1	1	0	1	1	1	1	1	0	0	0	1	1	1	0	0	0	1	1	1

Table S3. The species number of individuals of amphibians registered around Chengdu, Sichuan, China in 2019 and 2020.

Family	Genera	Species	Species number of individuals
Dicroglossidae	*Quasipaa*	*Quasipaa boulengeri*	28
	Fejervarya	*Fejervarya multistriata*	231
	Quasipaa	*Quasipaa spinosa*	9
Bufonidae	*Bufo*	*Bufo gargarizans*	154
Microhylidae	*Microhyla*	*Microhyla fissipes*	70
	Kaloula	*Kaloula rugifera*	26
Megophryidae	*Oreolalax*	*Oreolalax popei*	4
Rhacophoridae	*Polypedates*	*Polypedates megacephalus*	14
Ranidae	*Pelophylax*	*Pelophylax nigromaculatus*	93
	Odorrana	*Odorrana graminea*	6
	Odorrana	*Odorrana schmackeri*	16
	Rana	*Rana chaochiaoensis*	5
	Rana	*Rana chensinensis*	14
	Rana	*Rana omeimontis*	3
	Lithobates	*Lithobates catesbeiana*	2
	Hylarana	*Hylarana guentheri*	97
	Amolops	*Amolops mantzorum*	48
